# Novel Small Noncoding RNAs in Mouse Spermatozoa, Zygotes and Early Embryos

**DOI:** 10.1371/journal.pone.0044542

**Published:** 2012-09-12

**Authors:** Mitsuoki Kawano, Hideya Kawaji, Valérie Grandjean, Jafar Kiani, Minoo Rassoulzadegan

**Affiliations:** 1 RIKEN Omics Science Center, Yokohama, Kanagawa, Japan; 2 Graduate School of Dental Medicine, Hokkaido University, Sapporo, Hokkaido, Japan; 3 Inserm, U636, Nice, France; 4 Laboratoire de Génétique du Développement Normal et Pathologique, Université de Nice-Sophia Antipolis, Nice, France; Sun Yat-sen University, China

## Abstract

The recent discovery of a significant amount of RNA in spermatozoa contradicted the previously held belief that paternal contribution was limited to one copy of the genome. Furthermore, detection of RNA in sperm raised the intriguing question of its possible role in embryonic development. The possibility that RNAs may serve as epigenetic determinants was supported by experiments showing inheritance of epigenetic traits in mice mediated by RNA. We used high-throughput, large-scale sequencing technology to analyze sperm RNA. The RNA sequences generated were diverse in terms of length and included mRNAs, rRNAs, piRNAs, and miRNAs. We studied two small noncoding RNAs enriched in mature sperm, designated sperm RNAs (spR) −12 and −13. They are both encoded in a piRNA locus on chromosome 17, but neither their length (20–21 nt), nor their sequences correspond to known piRNAs or miRNAs. They are resistant to periodate-oxidation-mediated reaction, implying that they undergo terminal post-transcriptional modification. Both were detected in sperm and ovulated unfertilized oocytes, present in one-cell embryos and maintained in preimplantation stages, but not at later differentiation stages. These findings offer a new perspective regarding a possibly important role for gamete-specific small RNAs in early embryogenesis.

## Introduction

The discovery of significant amounts of RNA in the transcriptionally inert spermatozoon led to speculation regarding its possible role in embryonic development [1], [2]. Independently, the discovery of RNA-mediated inheritance of epigenetic traits in the mouse led us to the conclusion that sperm RNAs may act as transgenerational epigenetic determinants [3], [4], [5]. This prompted us to perform an in-depth evaluation of the spermatozoon RNA and, especially, the small noncoding (sncRNA) fraction. Previous knowledge was limited to the presence of microRNAs (miRNAs), whose functions in sperm remain open to question [6], [7]. We used deep sequencing to analyze the snc RNAs of mouse sperm. The same approach was recently applied by Krawetz et al. to the study of the major fractions of human sperm RNA, including miRNAs, Piwi-interacting RNA (piRNAs) and repeat-associated small RNAs [8]. Both piRNA and miRNAs are endogenous small RNAs, but piRNAs are distinct from miRNAs in their length (piRNA:∼24–31 nt; miRNAs: ∼21 nt) and expression patterns in that piRNAs are present in pachytene spermatocytes and round spermatids [9], while miRNAs have been discovered in a variety of species, cells and tissues at various development stages [10]. piRNAs often initiate with a 5′ uracil and contain 2′-*O*-methyl groups at their 3′ ends [11] and often found in clusters throughout the genome [12].

Our results identify two novel sncRNAs in sperm, also present in zygote and maintained in the embryo exclusively at the very early stage. Their size, nucleotide sequences, expression patterns, and genetic location make these RNAs distinct from known miRNAs and piRNAs.

## Results and Discussion

**Figure 1:**
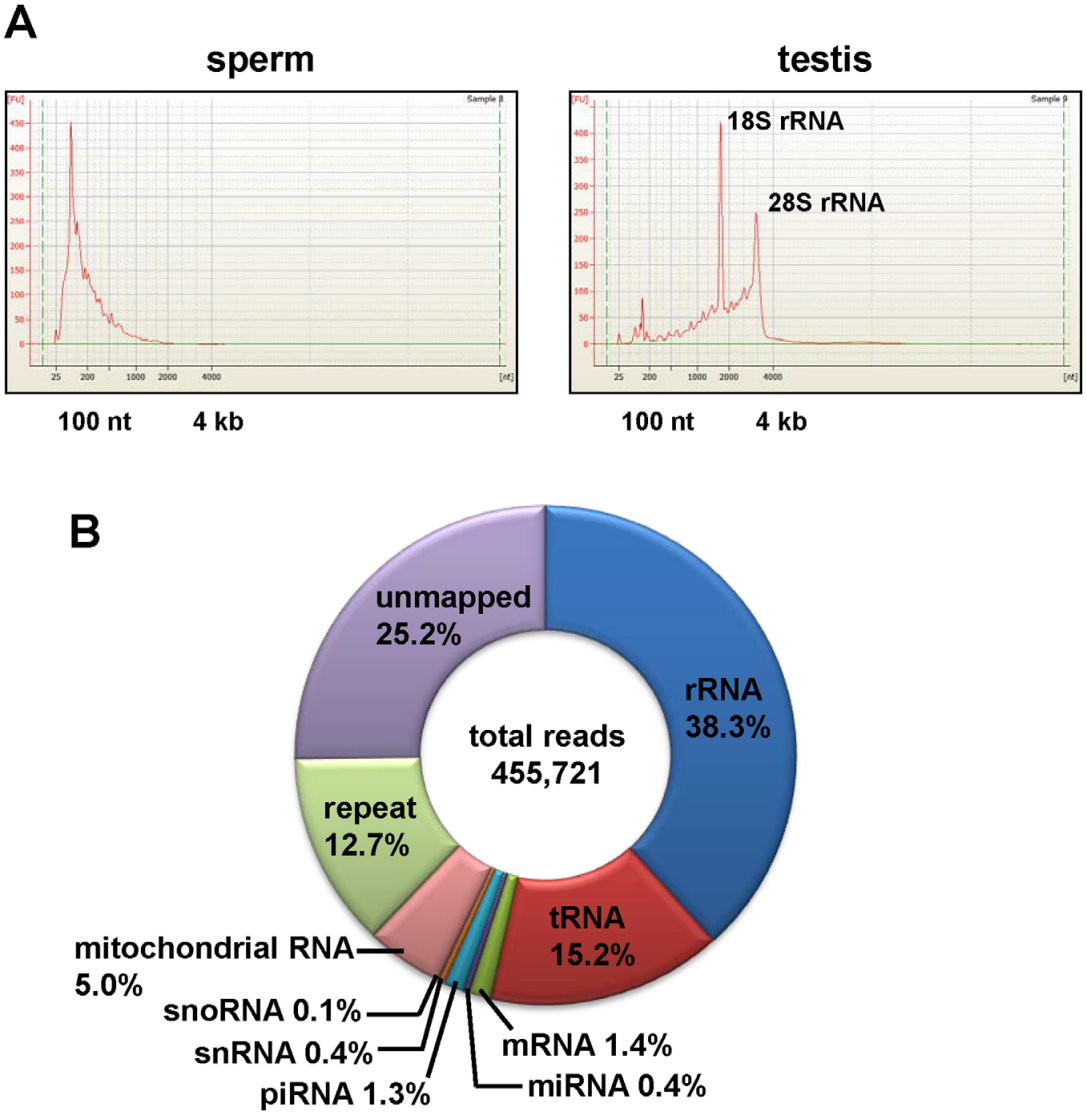
Size distribution of testis and sperm RNAs. Electropherograms were obtained using the RNA 6000 Nano Chip. **A.** RNA prepared from mouse sperm contained no or very low levels of long RNAs, including ribosomal RNAs (18S- and 28S-rRNA) compared with the total RNA from mouse testis **(B)**. **C.** Classification of all sequence reads in the mouse sperm total RNA library.

Sequencing of the small RNA fraction prepared from mouse sperm using a 454 sequencer resulted in 359,840 RNA sequences, ranging from 13 to 248 nt. As expected from previous studies [8], sperm RNA appeared as a complex mixture of break down products (Figure 1A) derived from long RNAs (rRNA, tRNA and mRNA) and small RNAs, including known miRNAs and piRNAs (Figure 1B).

**Figure 2:**
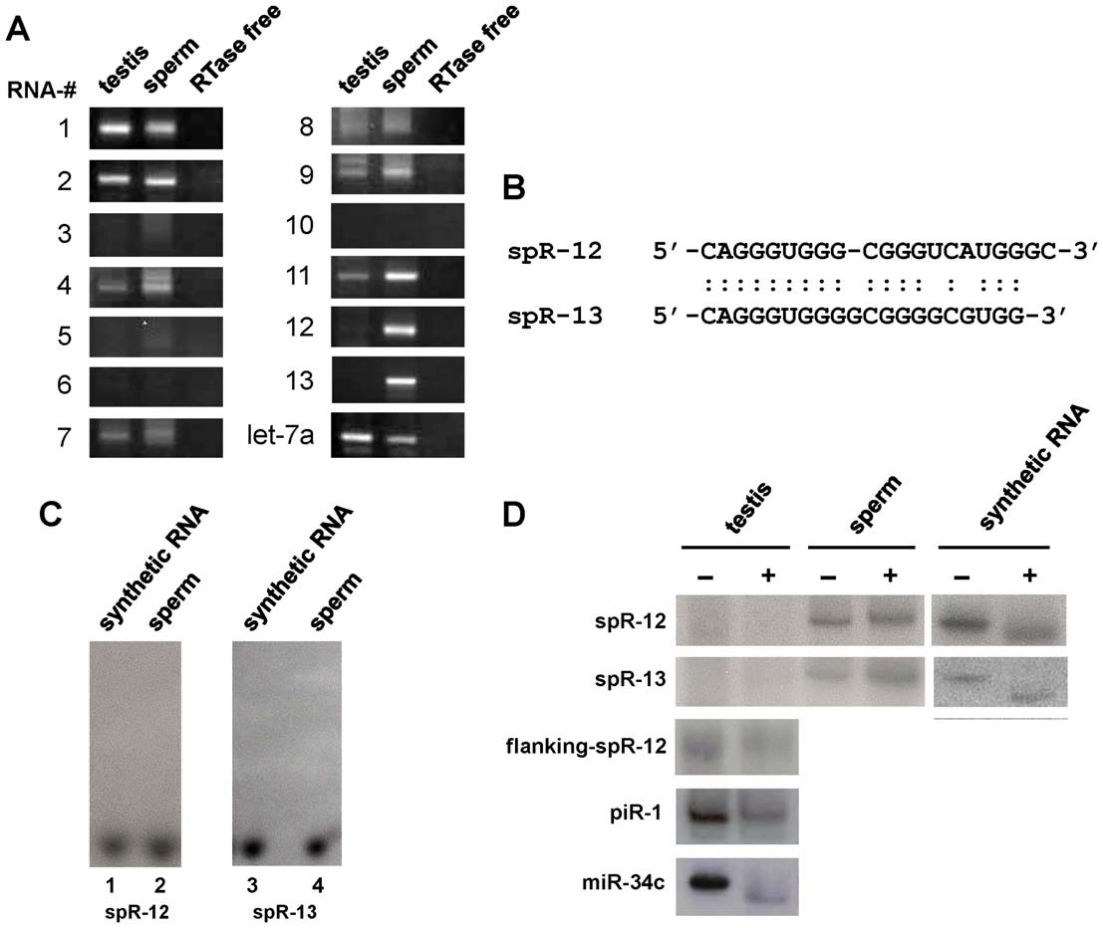
Expression analysis of thirteen sperm small RNAs. A. Poly(A)-tailed RT-PCR validation of 13 RNAs identified by deep sequencing. RNAs isolated from adult testis and sperm were polyadenylated. Reverse transcription was carried out using an RTQ primer, with or without reverse transcriptase. The cDNAs were amplified by PCR using a primer specific to each small RNA and an RTQ-UNIr universal primer (Table 5). The expected cDNA sizes for the RNAs were approximately 120 bp. The PCR products were electrophoresed on 3% (w/v) agarose gels and stained with ethidium bromide. The positive control was let-7a. **B.** spR-12 and -13 nucleotide sequences. **C.** Detection of spRs by Northern blot hybridization. Lanes 1 and 3: synthetic oligoribonucleotides with the sequences of spR-12 and -13, respectively; lanes 2 and 4∶1 µg of total sperm RNA; lanes 1 and 2 hybridized with spR-12-antisense probe, lanes 3 and 4 with spR-13-antisense probe (Table 5). **D.** Analysis of posttranscriptional modification of spRs termini. Northern blot analysis of testis, sperm samples and synthetic spRs, untreated (−) or treated (+) with the oxidation and β-elimination reagents. Only RNAs having both 2′ and 3′ hydroxyl groups react with NaIO_4_; β-elimination shortens a NaIO_4_-reacted RNA by one nucleotide, leaving a 3′ monophosphate terminus. NaIO_4_-reacted (β-eliminated) RNAs migrate faster in polyacrylamide gel electrophoresis than does the original untreated RNA. Both spRs, flanking-spR-12 (mmu_piR_032165) and piR-1 (mmu_piR_030365) lack either a 2′ or 3′ hydroxyl group, because they failed to react with NaIO_4_.

**Figure 3:**
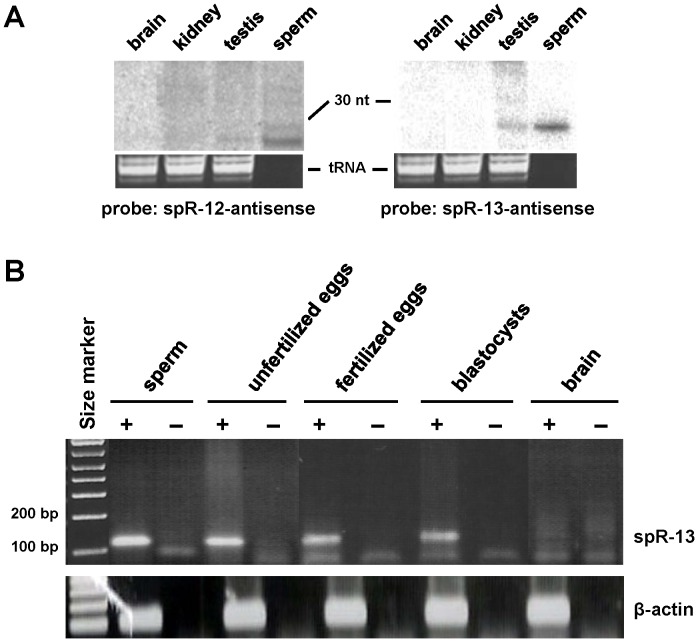
Expression of spR-12 and -13 RNAs in somatic and germinal cells. A. Northern blot analysis of testis, brain, and kidney RNA (10 µg of each) and 100 ng of total sperm RNA, which were hybridized with ^32^P-labelled antisense oligonucleotides. The loading control used was tRNA. The amount of sperm RNA was too low to be visualized by ethidium bromide staining. **B.** spR-13 expression in sperm, oocytes, embryos, and brain was analyzed by RT-PCR. Polyadenylation was performed either on total RNA isolated from sperm and brain, or directly on isolated oocytes and embryos without RNA purification. Reverse transcription was carried out using an RTQ primer, with or without reverse transcriptase. The cDNAs were amplified by PCR using a primer specific to spR-13 RNA and an RTQ-UNIr universal primer (Table 5). To validate the assay, each product was cloned and five independent clones were sequenced; all of the clones corresponded to the entire spR-13 sequence.

We then searched computationally for small RNAs that have stem-loop structure, and predicted 13 putative RNAs (see Materials and Methods). Nine of them were confirmed by a semiquantitative poly(A)-tailed RT-PCR method optimized for small RNAs [13] and two of them with higher expression levels in sperm (Figure 2A and Table 1), designated spR-12 and -13 which are highly similar (Figure 2B), were confirmed their existence and size by Northern blot analysis (Figure 2C). Both are resistant to periodate-oxidation-mediated reaction, implying that their termini contain post-transcriptional modifications (Figure 2D). We first selected for further analysis the 21-nt spR-12 RNA. Sequencing of the small RNA of sperm and total testis RNA using an Illumina GA sequencer confirmed its sequence, with a minority of nucleotide variants (Table 2), and its preferential accumulation in sperm (Table 3). The copy numbers of spR-12 in somatic and germ line cells were estimated by the stem-loop qRT-PCR method for quantification of small RNAs [14]. As shown in Table 4, all the tested somatic tissues were negative, while spR-12 was significantly accumulated in sperm. A lower level of expression in total testis RNA was first apparent at the age of two weeks, the time of entrance into meiosis, and these results were confirmed by Northern blot analysis (Figure 3A). Interestingly, spR-12 was also detected by the stem-loop qRT-PCR in ovulated unfertilized eggs, in one-cell embryos, and was maintained through the early developmental stages (Table 4). As also shown in Figure 3A and B, essentially identical results were obtained when the analysis was extended to spR-13, the related, but distinct RNA molecule detected by deep sequencing. Expression in sperm of spR-12 and -13 was confirmed by using the two sequencing techniques (454 and Illumina GA sequencers), by Northern blot analysis (Figure 2C and 3A) and by quantitative RT-PCR analysis followed by sequencing of the amplified product. Their unique stage specific expression in gametes and early embryo, Northern detection at the expected size and the correct sequences of PCR products make it highly unlikely that the two small RNAs could be products of a random degradation process.

**Table 1:** Novel small RNAs from the mouse sperm identified by deep sequencing.

Small RNA	Sequences (5′–3′)	Size(nt)	No. of reads	Location^a^	piRNA locus^b^	Stem-loop^c^	RT-PCR^d^
spR-1	AGG AAA CUG CCU CUC GGG GCA U	22	1	chr1 (+)	No	Yes	Yes
spR-2	CUG ACA GCA AGG CCU CUC CC	20	2	chr2 (+)	No	Yes	Yes
spR-3	ACU CCG GGC UGC UCG GGA GCC	21	1	chr4 (−)	No	Yes	No
spR-4	UGG GAA AGA CUC UGG GUC UCC U	22	2	chr4 (+)	No	Yes	Yes
spR-5	UGG CCU GGG CCU GGC AGU GGG C	22	1	chr6 (−)	No	Yes	No
spR-6	UCU GCC CUU CCC UCU GGA GAG U	22	1	chr7 (+)	No	Yes	No
spR-7	GUG UGU GCG UGU GUG GGC GC	20	1	chr8 (−)	No	Yes	Yes
spR-8	UGC CAG UGU GUG AGU GUG UG	20	1	chr8 (−)	No	Yes	Yes
spR-9	GUG CCC AGU CAU GUC CGG GGG CC	23	1	chr10 (+)	No	Yes	Yes
spR-10	CGU GGG ACC UUA GCG UUA UGC CG	23	1	chr13 (+)	No	Yes	No
spR-11	GAA CAG CCG AGU CCU UGC UGG U	22	1	chr13 (+)	No	Yes	Yes
spR-12	CAG GGU GGG CGG GUC AUG GGC	21	1	chr17 (−)	Yes	Yes	Yes
spR-13	CAG GGU GGG GCG GGG CGU GG	20	1	chr17 (+)	Yes	Yes	Yes
piRNA-locus-sR-1	UUC UCU GUG GGG UCC UGG CUG U	22	2	chr7 (+)	Yes	No	No
piRNA-locus-sR-2	CUG GGA CGG AUG GUG GGA GGG CA	23	1	chr17 (−)	Yes	No	No
piRNA-locus-sR-3	ACU GAG CCU UAU CAG GGU UC	20	2	chr5 (−)	Yes	No	No

a (+): plus strand, (−): minus strand.

b piRNA locus: mapped in piRNA locus.

c Stem-loop: potential pre-miRNA like secondary structure.

d RT-PCR: validated by poly(A)-tailed RT-PCR.

**Table 2:** Illumina GA sequencing reads of spR-12 sequences and variants.

Name	Sequence	Raw read counts	
Genome sequence	CAGGGTGGGCGGGTCATGGGCTTTGAG….	Sperm	Testis
spR-12	CAGGGTGGGCGGGTCATGGGC	374	2
spR-12 variant-1	CAGGGTGGGCGGGTCATGGGC**A**	224	7
spR-12 variant-2	CAGGGTGGGCGGGTCATGGGCTT	72	0
spR-12 variant-3	CAGGGTGGGCGGGTCATGGGCT	15	0
spR-12 variant-4	CAGGGTGGGCGGGTCATGGGC**AA**	10	1
spR-12 variant-5	CAGGGTGGGCGGGTCATGGGCT**A**	9	0
spR-12 variant-6	CAGGGTGGGCGGGTCATGGGC**G**	5	0
spR-12 variant-7	CAGGGTGGGCGGGTCATGGGC**AAA**	1	0
spR-12 variant-8	CAGGGTGGGCGGGTCATGGGC**C**	1	0

Bold type letters show nucleotide sequences which are not mapped on the genome.

**Table 3:** Sperm/testis ratio of read counts of spR-12 and flanking-spR-12.

Sequence	Sperm	Testis	Sperm/testis ratio
spR-12	59.9	0.4	150
flanking-spR-12	35.9	69.2	0.5

Read counts of each sequence were normalized to reads per million (RPM).

**Table 4:** Quantitative PCR determination of spR-12 in somatic and germ line cells.

Cell type	Relative expression level
Kidney	<1*
Brain	<1*
Lung	<1*
Liver	<1*
Testis (1-week old)	<1*
Testis (2-week old)	5±2*
Testis (3-week old)	95±10*
Testis (adult)	231±33*
Sperm	100,000±70*
Ovulated oocyte	21±10**
Fertilized eggs	50±18**
Blastocyst	22±11**

* Expression relative to that of miR-16.

** Expression relative to actual number of cells. Serial dilutions of synthetic spR-12 RNA were used to generate a standard curve of the log of template quantity versus threshold cycle (C_(t)_). The concentration of RNA ranged from 10–10^6^ initial copies. A minimum of eight oocytes and/or embryos was used in each experiment. Each value corresponds to three or four independent experiments, performed in duplicate. The analysis for spR-13 was not done as it was not possible to synthesize the spR-13 RT-primer.

**Table 5:** Oligonucleotides used in this study.

Oligonucleotide name	Sequence (5′–3′)	Assay employed in
RTQ primer	CGA ATT CTA GAG CTC GAG GCA GGC GAC ATG GCT GGC TAG TTAAGC TTG GTA CCG AGC TCG GAT CCA CTA GTC C(T)_25_V N	polyA-RT-PCR
RTQ-UNIr	CGA ATT CTA GAG CTC GAG GCA GG	polyA-RT-PCR
spR-1	AGG AAA CTG CCT CTC GGG GC	polyA-RT-PCR
spR-2	CTG ACA GCA AGG CCT CTC	polyA-RT-PCR
spR-3	ACT CCG GGC TGC TCG GGA G	polyA-RT-PCR
spR-4	TGG GAA AGA CTC TGG GTC TC	polyA-RT-PCR
spR-5	TGG CCT GGG CCT GGC AGT GG	polyA-RT-PCR
spR-6	TCT GCC CTT CCC TCT GGA GA	polyA-RT-PCR
spR-7	GTG TGT GCG TGT GTG GGC	polyA-RT-PCR
spR-8	TGC CAG TGT GTG AGT GTG	polyA-RT-PCR
spR-9	GTG CCC AGT CAT GTC CGG GGG	polyA-RT-PCR
spR-10	CGT GGG ACC TTA GCG TTA TGC	polyA-RT-PCR
spR-11	GAA CAG CCG AGT CCT TGC TG	polyA-RT-PCR
spR-12	CAG GGT GGG CGG GTC ATG G	polyA-RT-PCR
spR-13	CAG GGT GGG GCG GGG CG	polyA-RT-PCR
flanking-spR-12	TGA TAG CTG AGC TCT GAG	polyA-RT-PCR
flanking-spR-13	TGT CAT TGA TCC AGG CAA TG	polyA-RT-PCR
let-7a	TGA GGT AGT AGG TTG TAT AG	polyA-RT-PCR
piRNA-locus-sR-1	TTC TCT GTG GGG TCC TGG CT	polyA-RT-PCR
piRNA-locus-sR-2	CTG GGA CGG ATG GTG GGA GG	polyA-RT-PCR
piRNA-locus-sR-3	TTA CTG AGC CTT ATC AGG GT	polyA-RT-PCR
piR12-F	CCC TCA ACA AAT AGT CTG TCT	strand-specific RT-PCR
piR12-R	TGG AAG CTT CCT TCC CTG TC	strand-specific RT-PCR
piR13-F	TCT CCC TTC TGT TTT CCG TG	strand-specific RT-PCR
piR13-R	CCC TGA GCA GTC ACA CTT TG	strand-specific RT-PCR
LINE 1-sense	AGT GCA GAG TTC TAT CAG ACC TTC	strand-specific RT-PCR
LINE 1-antisense	TCC AGC TAC TTC ACC GAA GCT GT	strand-specific RT-PCR
beta-actin-sense	CCA CCA CAG CTG AGA GGG AA	strand-specific RT-PCR
beta-actin-antisense	AGC CAC CGA TCC ACA CAG AG	strand-specific RT-PCR
spR-12-antisense	GCC CAT GAC CCG CCC ACC CTG	Northern blotting
spR-13-antisense	CCA CGC CCC GCC CCA CCC TG	Northern blotting
flanking-spR-12-antisense	ACT ACC TAC TCA GAG CTC AGC TAT CA	Northern blotting
piR-1-antisense	AAA GCT ATC TGA GCA CCT GTG TTC ATG TCA	Northern blotting
miR-34c-antisense	GCA ATC AGC TAA CTA CAC TGC CT	Northern blotting

For the oligonucleotide, RTQ primer, V represents the nucleotides A, C, or G; N indicates that the nucleotide is equivalent to A, C, G, or T.

**Figure 4:**
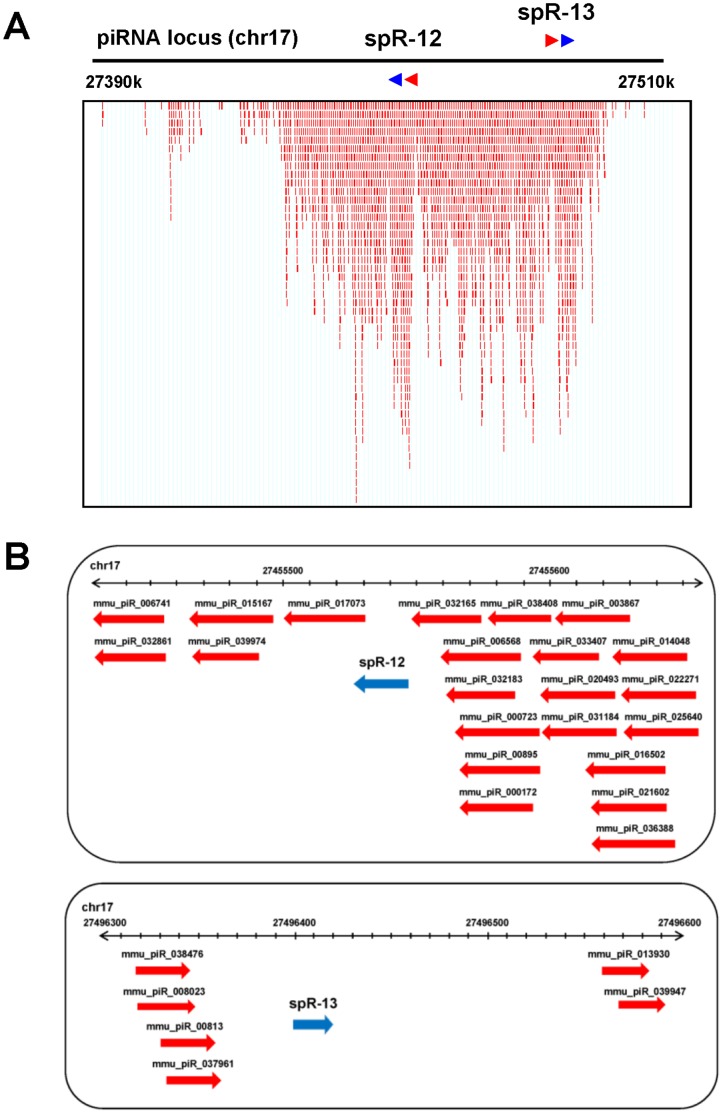
Genomic location of spR-12 and -13 and the proximal piRNAs. **A.** The piRNA clusters that map to spR-12 and -13 maps. Sequences of spR-12 and -13 are shown as blue arrowheads, and their known adjacent piRNAs are indicated as red arrowheads above the box. The size of the arrowhead is not scaled. The red lines in the box indicate piRNAs registered in piRNABank (http://pirnabank.ibab.ac.in/). **B.** Map positions of spR-12 and -13 reads and known piRNAs in piRNABank.

**Figure 5:**
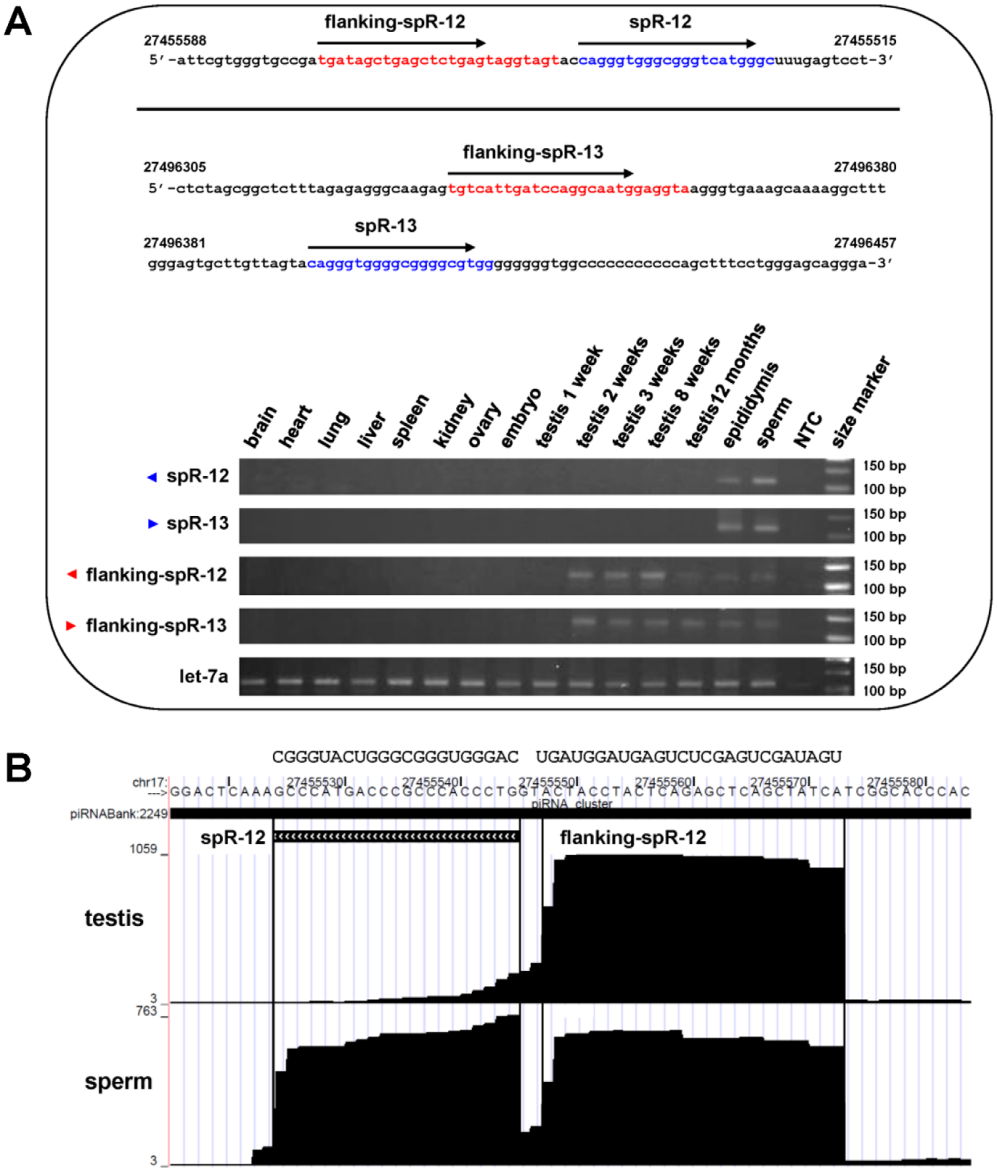
Expression profiling of spR-12 and -13 and the proximal piRNAs. **A.** RT-PCR analysis of spR-12 and -13 and their flanking piRNAs in different mouse tissues. The piRNAs adjacent to spR-12 (mmu_piR_032165) and -13 (mmu_piR_037961) and the spR-12 and -13 sequences are indicated in red and blue, respectively. Numbers are the nucleotide coordinates of the genomic DNA. Primers specific to each small RNA are shown as blacks arrows. RT-PCR was performed with a specific primer and RTQ-UNIr universal primers (Table 5). The PCR products were analyzed on 3% (w/v) agarose gels and contained the expected 120 bp fragment. The positive control was let-7a. **B.** Short RNA reads mapped in the spR-12 locus and the adjacent piRNA mum_piR_032165. X axis indicates the genomic coordinates and Y axis the number of sequence reads. Location of the spR-12 and piRNA sequences is indicated with the genomic coordinates and number of read counts.

Both spRs are encoded in a region of approximately 40 kb, within a piRNA cluster on chromosome 17, and are oriented divergently, in the same way as the proximal piRNAs (Figure 4A and 4B). Analysis by poly(A)-tailed RT-PCR indicated that expression of the flanking piRNAs reached a peak at earlier differentiation stages in male germ cells compared with those of spR-12 and -13 (Figure 5A). Read counts in the border region between spR-12 and the adjacent piRNA (piR-032165) clearly show equivalent numbers of reads of the piRNA and spR sequences in sperm RNA, however there are no or very little spR-12 reads in testis RNA (Figure 5B).

**Figure 6:**
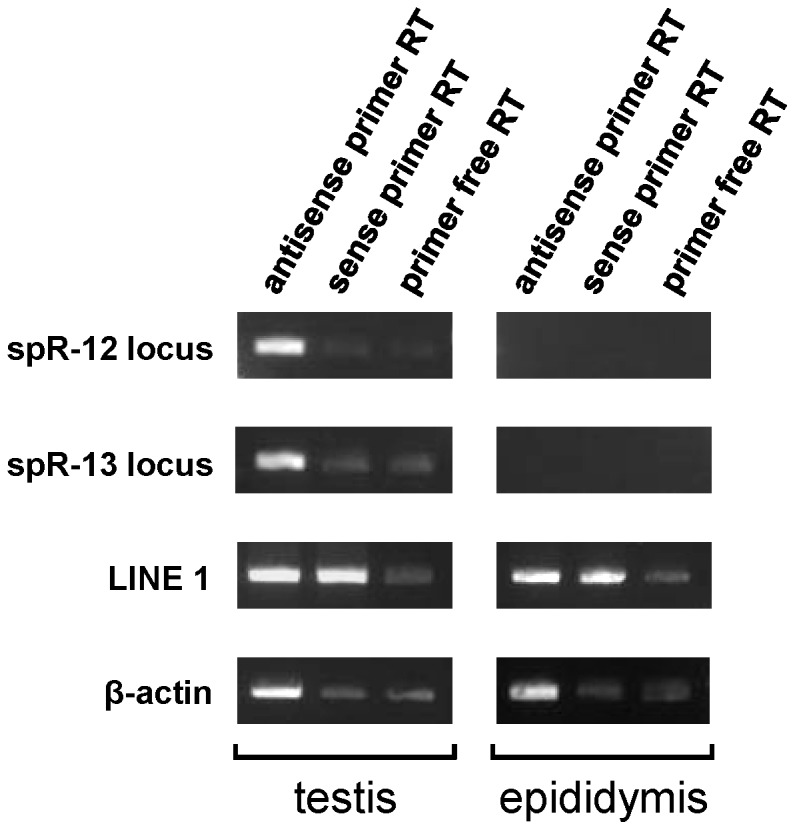
Search for double-stranded piRNA precursors of spR-12 and -13 by strand-specific RT-PCR. Total RNA from adult mouse testes and epididymis was reverse-transcribed with a primer with an antisense sequence, with a primer with a sense sequence, and with no primer. PCR was conducted using gene-specific primers (Table 5). The product was 301 bp for the spR-12 locus, and 351 bp for the spR-13 locus, whose nucleotide sequences are shown in Figure 7. LINE 1 and β-actin were used as positive and negative controls, respectively.

**Figure 7:**
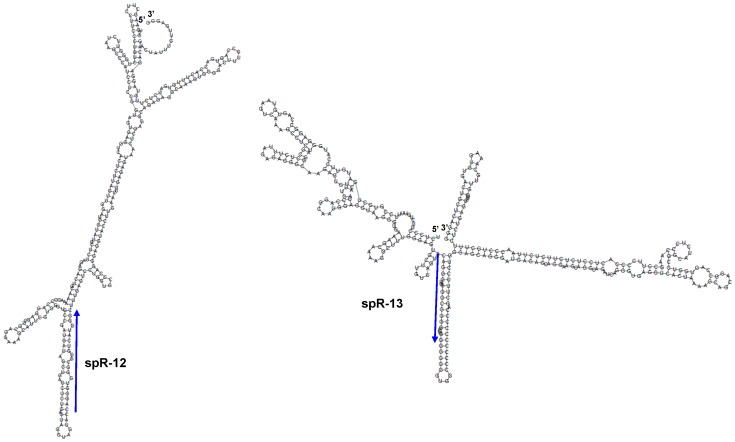
Predicted secondary structures of spR-12 and -13 regions. Positions in the mouse genome used for this analysis were chr17∶27455400–27455700 for spR-12 and chr17∶27496250–27496600 for spR-13. Position of the cDNA read sequences are shown by the blue arrows. The structure shown is the most stable configuration predicted by ‘RNAfold’ [20].

The piRNAs were reported to generate endogenous siRNAs (∼21 nt) from the long double-stranded RNAs (dsRNAs) expressed from retrotransposons and pseudogenes in mouse oocytes [15], [16]. Therefore, spR-12 and -13 might also be derived from long dsRNAs. However, no transposable element or pseudogene with related sequences is present at either locus. We did not detect antisense transcription using strand specific RT-PCR (Figure 6). Prediction of the secondary structures of the proximal ∼300 nt regions did not show long stretches of dsRNA. Therefore, we hypothesize that spR-12 and -13 may be derived from the piRNA precursor transcript and cleaved from stem-loop structured RNA, instead of long dsRNA like siRNA production (Figure 7). Their sequences do not match those of any known miRNA or piRNA (miRBase: http://mirbase.org/, piRNABank: http://pirnabank.ibab.ac.in) [17].

In summary, we showed that two small noncoding RNAs, distinct from typical micro- and pi-RNAs, are present only in the gametes and maintained during the early stages of development. Their accumulation, zygotic transfer and maintenance during early development raise a number of intriguing questions for further studies. The event from piRNA expression to spR accumulation indicates a novel pathway for small RNA biogenesis during spermatogenesis. Maintenance of spRNAs during the pre-implantation period and their disappearance at the blastocyst stage suggest that they may exert important function(s) in early embryonic development. Future genetic and biochemical studies will hopefully help to determine the roles of small RNAs during early embryogenesis.

## Materials and Methods

### Ethics Statement

This research involving animals was conducted according to the approval of the Animal Research Ethics Committee of RIKEN Yokohama Institute. The approved permit number for this study is “22–033”.

### Isolation of Mouse Sperm RNA

Spermatozoa were collected from 74 mouse caudae epididymides of strain C57BL/6. Motile spermatozoa were washed twice in MEM buffer (1 mM Na pyruvate, 0.5 mM EDTA, 50 U/ml Penicillin, 50 µg/ml Streptomycin, and 0.1% BSA) by centrifugation. Sperm pellets were resuspended in phosphate-buffered saline (PBS), followed by centrifugation. The pellets were washed twice in storage buffer (50 mM HEPES buffer at pH 7.5, 10 mM NaCl, 5 mM Mg acetate, and 25% glycerol), then stored at −80°C. Subsequent to storage, the samples were thawed, and washed twice in cell lysis buffer (0.1% sodium dodecyl sulfate (SDS) and 0.5% Triton X-100 in H_2_O). This procedure generates an essentially pure population of spermatozoa [18]. Non sperm cell contamination was checked by microscopy. Total RNA was extracted from the purified sperm using TRIzol (Invitrogen, Carlsbad, CA, USA) and Acid-Phenol:Chloroform (Ambion, Austin, TX, USA), according to the manufacturer’s instructions. The concentration and integrity of the total RNA samples were evaluated using a NanoDrop ND-1000 spectrophotometer (Thermo Fisher Scientific Inc., Wilmington, DE, USA) and a 2100-Bioanalyzer with the RNA 6000 Nano Chip (Applied Biosystems, Carlsbad, CA, USA) shown in Figure 1, respectively.

### Small RNA Library Construction and Deep Sequencing

A small RNA cDNA library was generated from ∼0.8 µg of total RNA, as previously described [19], but without cloning the library into a plasmid for massively parallel pyrosequencing using a 454 genome sequencer (Roche, Basel, Switzerland). For deep sequencing using an Illumina GA sequencer (Illumina, San Diego, CA, USA), we constructed cDNA libraries of mouse sperm and testis total RNA using the Small RNA Samples Prep Kit (Illumina), according to the manufacturer’s instructions. The sequencing results are archived in the DDBJ Sequence Read Archive (DRA) database (accession number: DRA000621).

### Classification of Small RNAs

First, we aligned the small RNA sequences to known RNA sequences retrieved from public databases. We accepted only complete matches using nexAlign (version 1.3.5) (http://genome.gsc.riken.jp/osc/english/dataresource/). The mature and star sequences of miRNAs were downloaded from miRbase (version 15.0); RefSeq mRNA sequences were from the UCSC Table Browser; and piRNA, snoRNA, and snRNA were from NONCODE (version 2). The tRNA sequences were prepared from the genome sequence with an additional CCA at the 3′-end, which was based on RefSeq annotation of NCBI Build 37 (mouse). The remaining small RNA sequences, which did not match to the RNA sequences above, were aligned with the mouse genome sequence (NCBI Build 37 or mm9), and their genomic coordinates were compared with the repeat masker track in the UCSC genome browser database. The small RNA sequences were classified based on their matches to known RNA sequences or overlaps on the genomic coordinates.

### Computational Exploration of Novel Small RNAs in Sperm

We aligned the sequenced 20 to 23-nucleotide small RNA sequences, which were neither miRNA nor rRNA, to the genome sequence. After screening out RNAs that mapped to two or more loci, we identified 39 loci as potential candidates. The putative secondary structures of their proximal regions were predicted by the RNAfold (http://rna.tbi.univie.ac.at/cgi-bin/RNAfold.cgi) [20], and the results were inspected manually to check if they were hairpin-like structures harboring the small RNA sequence as one strand of its stem. The secondary structures were created by mfold (http://mfold.bioinfo.rpi.edu/) [21], followed by manual editing.

### Semiquantitative Poly(A)-tailed RT-PCR

The PCR-based detection of small RNAs was carried out with 2 µg of total RNA samples isolated from various adult mouse tissues (8–9 weeks), testes at various ages (1–8 weeks, and 12 months), and embryo (15 days), and 0.7 µg of total RNA for sperm (9 weeks). The RNA was polyadenylated using a poly(A) Tailing Kit (Ambion) and used in the synthesis of small RNA cDNA with PrimeScript II RTase (Takara Bio, Ohtsu, Shiga, Japan) and 2.5 µM of RTQ primer [13]. Individual RNAs were detected by PCR using AccuPrime Taq DNA SuperMix I (Invitrogen) and an RTQ-UNIr primer with gene-specific primers (Table 5).

### Northern Blot Analysis

Total RNA (10 µg) from testis or 1 µg from sperm samples and synthetic spR-12 and -13 oligoribonucleotides (∼6×10^7^ molecules each) were denatured for 5 min at 65°C in loading buffer containing 50% formamide, separated on 12% polyacrylamide/8 M urea gels, and then transferred to Nytran N membranes (Schleicher & Schuell, Germany) by electroblotting. The RNAs were cross-linked to the membrane by exposure to UV light. The 5′ oligonucleotide was end-labeled with [γ -^32^P] ATP and T4 polynucleotide kinase. The membranes were hybridized with ^32^P-end-labelled oligonucleotides for 18 hours at 42°C in hybridization buffer (0.25 M Sodium Hydrogen phosphate, 1 mM EDTA, 7% SDS) and washed 3×10 min with 2× SSC, 0.1% SDS at 42°C and 2×10 min with 0.1% SSPE, 0.1% SDS at 42°C. The hybridization signals were visualized using a PhosphorImager (Molecular Dynamics).

### Periodate-oxidation-mediated Reaction and β-elimination

Total RNA (12 µg) from testis or 2 µg from sperm samples were subjected to periodate-oxidation-mediated reaction and β-elimination as described [22]. NaIO_4_ reaction was performed by adding total testis and sperm RNA in water to 4 µl of 5× borate buffer (148 mM borax,148 mM boric acid, pH 8.6) and 2.5 µl of freshly dissolved 200 mM NaIO_4_ and incubating for 10 min at room temperature. 2 µl of glycerol was added to quench unreacted NaIO_4_ and incubated for an additional 10 min at room temperature. Samples were dried by centrifugation under vacuum for 1 hour at room temperature, then dissolved in 50 µl of 1× borax buffer (30 mM borax and 30 mM boric acid, 50 mM NaOH, pH 9.5) and incubated for 90 min at 45°C. 20 µg of glycogen was added, the RNAs were precipitated at −70°C for 1 hour, the precipitate samples collected by centrifugation and then dissolved in the denaturing gel loading buffer and ran in 20% polyacrylamaid gel containing 7 M urea. Synthetic spR-12 and -13 oligoribonucleotides, piR-1 [23] and miR-34c were used as controls for the β-elimination reaction. Northern blot hybridization using Hybond N+ membrane (GE Healthcare) was performed with antisense spR-12, spR-13, flanking-spR-12, piR-1 and miR-34c synthetic oligonucleotides as probes (Table 5)**.**

### Stem-loop Quantitative RT-PCR Determination of Small RNAs

Stem-loop quantitative RT-PCR reactions [14] were performed using the Custom TaqMan Small RNA Assays Kit (Applied Biosystems), following the manufacturer’s instructions, with slight modifications. Briefly, reverse transcription was performed on either total RNA isolated from sperm or tissues, or directly on 6–8 isolated embryos without RNA purification but with RNA stem-loop specific primers. The stem-loop structure of the RT-primer provided specificity only for the mature small RNA target, and formed an RT primer/mature RNA-chimera that extended the 5′ end of the RNA. The resulting longer RT amplicon presents a template amenable to standard real-time PCR.

### Strand-specific RT-PCR

DNase-treated total RNA (0.5 µg) from adult testis and epididymis was reverse-transcribed using PrimeScript II RTase (Takara) with gene-specific RT-primers (Table 5). The piRNA precursors, a protein-coding gene (β-actin), and a retrotransposon (LINE 1) were amplified using PrimeSTAR GXL DNA polymerase (Takara) with gene-specific sense and antisense primers (Table 5), according to the manufacturer’s instructions.
