# Correction: Novel Small Noncoding RNAs in Mouse Spermatozoa, Zygotes and Early Embryos

**DOI:** 10.1371/annotation/68f2e49d-f789-4d5c-a965-5805358cc454

**Published:** 2013-05-14

**Authors:** Mitsuoki Kawano, Hideya Kawaji, Valérie Grandjean, Jafar Kiani, Minoo Rassoulzadegan

The version of Figure 1 in the article was incomplete. The complete, correct version of Figure 1 is available here: 

**Figure:**
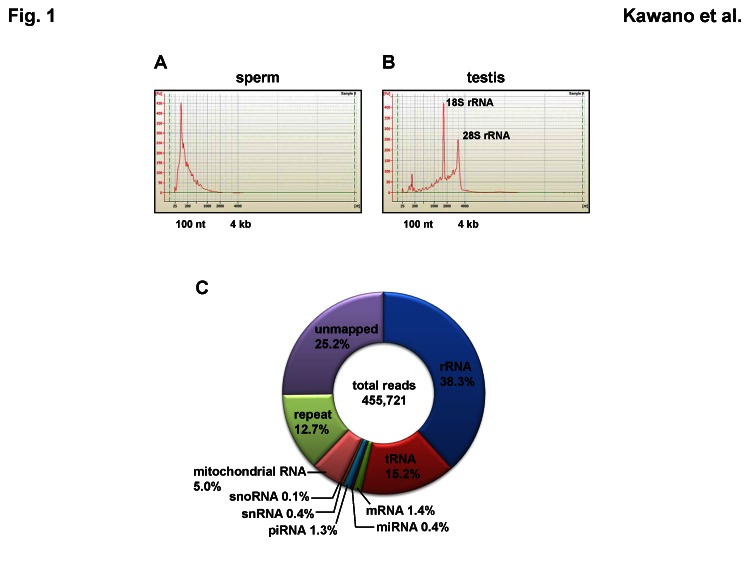


References 12 and 13 were combined in the published article. This means that each Reference above 12 should be one number higher. 

